# Wet-Oxidation-Assisted Chemical Mechanical Polishing and High-Temperature Thermal Annealing for Low-Loss 4H-SiC Integrated Photonic Devices

**DOI:** 10.3390/ma16062324

**Published:** 2023-03-14

**Authors:** Xiaodong Shi, Yaoqin Lu, Didier Chaussende, Karsten Rottwitt, Haiyan Ou

**Affiliations:** 1 Department of Electrical and Photonics Engineering, Technical University of Denmark, rsteds Plads, Building 343, 2800 Lyngby, Denmark; 2 Université Grenoble Alpes, CNRS, Grenoble INP, SIMaP, 38000 Grenoble, France

**Keywords:** silicon carbide, integrated photonics, chemical mechanical polishing

## Abstract

Silicon carbide (SiC) has become a promising optical material for quantum photonics and nonlinear photonics during the past decade. In this work, we propose two methods to improve the 4H-SiC thin film quality for SiC integrated photonic chips. Firstly, we develop a wet-oxidation-assisted chemical mechanical polishing (CMP) process for 4H-SiC, which can significantly decrease the surface roughness from 3.67 nm to 0.15 nm, thus mitigating the light scattering loss. Secondly, we find that the thermal annealing of the 4H-SiC devices at 1300 °C can help to decrease the material absorption loss. We experimentally demonstrate that the wet-oxidation-assisted CMP and the high-temperature annealing can effectively increase the intrinsic quality factor of the 4H-SiC optical microring resonators.

## 1. Introduction

Silicon carbide (SiC) has been widely used in power electronics, thanks to its excellent electronic properties, including high breakdown electric field, high saturated electron drift velocity, and wide bandgap [1,2]. It is also a choice for temperature sensors [3,4]. Meanwhile, SiC also owns many outstanding optical properties, making this material very promising for making photonic and opto-electronic devices [5,6,7,8,9]. SiC has a wide window of transparency, from near ultraviolet to mid-infrared wavelength range, due to the wide bandgap. Compared to other wide-bandgap optical materials, SiC has high refractive index, enabling tight confinement of the lightwave. With strong material-based second- and third-order nonlinearities, SiC allows efficient wavelength conversion, such as four-wave mixing, Raman amplification, frequency comb generation, and second-harmonic generation [10,11,12,13,14]. Crystalline SiC holds various of intrinsic optically addressable color centers, which can be potentially applied to make integrated single-photon sources [15,16]. Leveraging these excellent optical properties and the advantages of the complementary metal-oxide-semiconductor (CMOS) technology, SiC is an emerging optical material for quantum photonics and nonlinear photonics [17].

The light propagation loss is one of the most important features for the integrated photonic platforms. Light circulating in the microring with a small footprint enables strong nonlinearity enhancement. Low-loss microring resonators can especially benefit nonlinear optical applications in the 4H-SiC-on-insulator (4H-SiCOI) integrated platforms. For example, four-wave mixing, frequency comb generation, and second-harmonic generation in the 4H-SiC microrings can exhibit much lower power threshold and higher conversion efficiency [18]. The loss usually comes from the scattering loss and the material absorption.

The scattering loss is mainly induced by the surface roughness. Hence, a smooth top surface of the photonic chips is highly desired. Polishing is an effective way to improve the surface roughness of the chips. SiC is a very hard material with a Mohs hardness of 9.3, which is only a little softer than diamond, with Mohs hardness of 10 [19]. It is harder than most of the other materials, for example, Si and SiO${}_{2}$, with Mohs hardness of 6.5 and 5.5, respectively. Because the material can only be polished by the abrasive with higher hardness, the diamond is mostly used to mechanically polish SiC. However, such mechanical polishing can create scratches and subsurface damages on the chip surface [20,21]. Chemical mechanical polishing (CMP) is a surface smoothing method, combining both chemical reaction and mechanical polishing. The chemical reaction is able to change the properties of the SiC surface and to make the surface softer. The abrasive, which is harder than the new surface and softer than SiC, can be used for polishing. Hence, only the soft surface is polished, and at the same time, no scratches or subsurface damages are created on SiC. SiC also has high chemical inertness. It can be slowly oxidized by strong oxidizers, such as O${}_{2}$, H${}_{2}$O${}_{2}$, and KMnO${}_{4}$, and can react with hot HF, H${}_{3}$PO${}_{4}$, NaOH, and KOH solutions [22]. For the CMP of SiC, the chemical reaction is to oxidize SiC to form a soft SiO${}_{2}$ layer on top of SiC, and then this layer can be polished with corrosive agents and abrasives with hardness between SiO${}_{2}$ and SiC thin films. For example, colloidal SiO${}_{2}$ and CeO${}_{2}$, of which the hardness is 6.5–7 and 6, respectively, can be used as the abrasive for polishing [23]. In general, there are mainly four CMP methods for SiC, which are wet-chemical-assisted CMP, electro-CMP, plasma-assisted CMP, and thermal-oxidation-assisted CMP [24,25,26,27,28]. For the wet-chemical-assisted CMP, the SiC surface is oxidized by strong oxidizers, such as H${}_{2}$O${}_{2}$ and KMnO${}_{4}$, but the oxidation rate of SiC at room temperature is quite low, and these corrosive chemicals are dangerous and not environmentally friendly. Electro-CMP and plasma-assisted CMP help to improve the surface reaction, but require additional setup hybrid in the standard CMP machine, such as electrical components and plasma generators. In the thermal-oxidation-assisted CMP, SiC is thermally oxidized in the furnace, and then polished by the abrasives, which does not need any strong oxidizing or corrosive wet agents in the chemical process, and the polishing process can be performed in the CMP machine easily. A method that combines dry oxidation of SiC at 1100 °C for 4 h and polishing with CeO${}_{2}$ abrasive slurry, obtaining surface roughness of 0.10 nm, has been reported [29]. However, dry oxidation has relatively low oxidizing rate, and during dry oxidation of SiC, a carbon layer is formed on top of the oxide [29], which cannot be dissolved in HF acid together with the oxide layer, so it requires extra processes before polishing the interface, such as plasma ashing.

In order to efficiently obtain 4H-SiC uniform thin films and fabricate 4H-SiCOI integrated platforms, the ion-cut method is usually applied, similar to the smart-cut technique to form SOI stacks [7]. In this process, hydrogen ions are implanted into the 4H-SiC bulk wafers to form a defect layer beneath the surface. After bonding the SiC wafer to a SiO${}_{2}$-Si wafer, an annealing process is used to vaporize the hydrogen ions and produce hydrogen bubbles to split the 4H-SiC thin film from the bulk wafer. However, it is found that photonic devices in such 4H-SiCOI chips suffer from a high material absorption induced light propagation loss [12]. The reason is considered to be plenty of crystal lattice damages, generated during the hydrogen ion implantation [30]. However, there is no any effective solution to solve the problem.

In this work, we develop a wet-oxidation-assisted CMP process to polish 4H-SiC chips and achieve a low surface roughness. We fabricate optical microring resonators on the 4H-SiC chips with and without polishing, and verify that the light propagation loss is largely reduced in the polished chip. We also find that the crystal lattice damages in the 4H-SiC thin film induced by the ion implantation can be partially recovered during the high temperature annealing, which helps to reduce light propagation loss.

## 2. Wet-Oxidation-Assisted Chemical Mechanical Polishing

A wet-oxidation-assisted CMP process is developed to smooth the 4H-SiC chips. The CMP machine, the polishing pad, and the abrasive slurry that we use are Logitech CM62 Orbis Chemical Mechanical Polisher, polyurethane pad, and alkaline colloidal silica slurry with an average diameter of 60 nm, respectively. The 4H-SiC chips are cut to be 2 cm × 2 cm for preparation. Firstly, the 4H-SiC chip is wet oxidized for 1 h at 1100 °C to generate a SiO${}_{2}$ thin film. Secondly, the SiO${}_{2}$ thin film can either be removed by 5% HF directly or be polished by the colloidal silica abrasive slurry with a fast removing rate. It has been demonstrated there is an intermediate layer between SiC and SiO${}_{2}$ generated during oxidation, which contains Si-C-O [28]. This intermediate layer cannot be dissolved by HF acid at room temperature, but can be polished by the colloidal silica.

**Figure 1:**
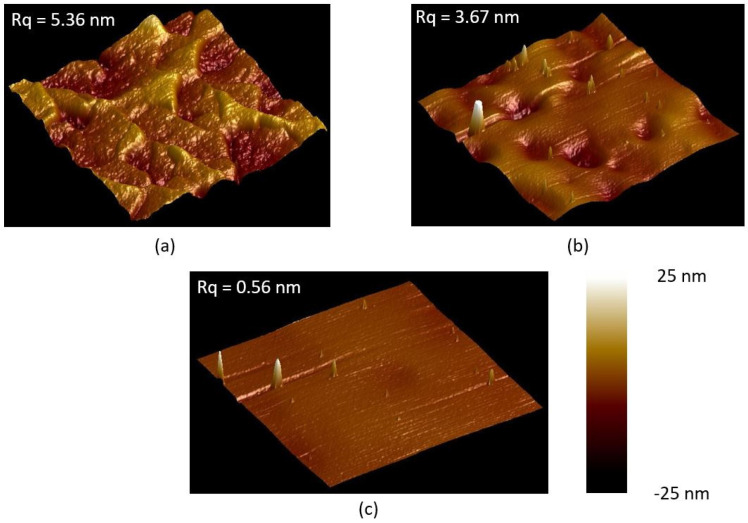
AFM images with 1 µm × 1 µm measurement fields of the 4H-SiC chip surface (**a**) without polishing, (**b**) with mechanical polishing by colloidal silica, and (**c**) with wet-oxidation-assisted CMP.

Figure 1a shows the atomic force microscopy (AFM) image of the initial surface topography of the 4H-SiC chip, which has a root-mean-square (RMS) roughness of 5.36 ± 0.41 nm. Figure 1b shows the AFM image of the surface topography of the 4H-SiC chip after mechanical polishing by the colloidal silica abrasive slurry. As can be seen, the surface becomes a little smoother than the initial chip, but still has a high RMS roughness of 3.67 ± 0.23 nm. It is because there is a native oxide layer formed on the top of the initial 4H-SiC chip surface, which can be polished by colloidal silica. However, after polishing the native oxide, colloidal silica cannot further polish the 4H-SiC due to its low hardness. Figure 1c shows the AFM image of the surface topography of the 4H-SiCOI chip after the wet-oxidation-assisted CMP. The surface becomes much smoother than the initial surface or the mechanically polished surface, and the RMS roughness is greatly decreased down to 0.56 ± 0.13 nm. Moreover, no scratches or subsurface damages are observed.

**Figure 2:**
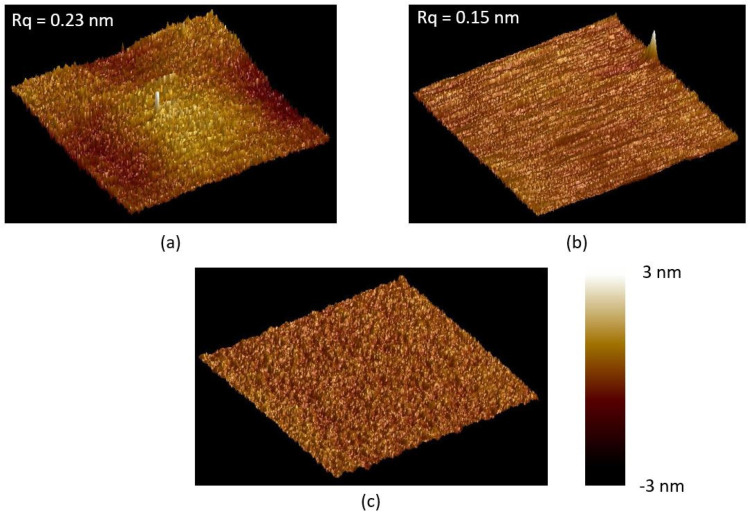
AFM images with 1 µm × 1 µm measurement fields of the 4H-SiC chip surface with (**a**) twice and (**b**) thrice wet-oxidation-assisted CMP processes, and (**c**) post oxidation after the third CMP.

**Figure 3:**
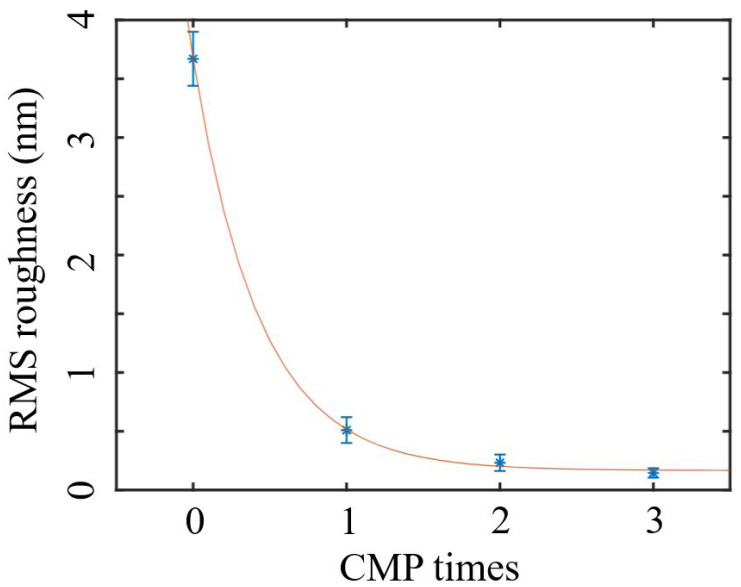
Surface roughness of the 4H-SiC chip versus the number of the wet-oxidation-assisted CMP processes.

We also find the surface roughness after wet-oxidation-assisted CMP is related to the initial surface roughness before polishing. A second and a third CMP are tested on the same chip. Figure 2a,b show the AFM images of the surface profiles of the 4H-SiCOI chip after the second and the third CMP processes, which result in lower RMS roughness of 0.23 ± 0.07 nm and 0.15 ± 0.04 nm, respectively. Meanwhile, the standard deviation also becomes smaller with more CMP processes. We finally achieve a rather low standard deviation of 0.04 nm within the 2 cm × 2 cm area, indicating that the chip surface has very good uniformity. The surface roughness as a function of the cycles of the wet-oxidation-assisted CMP processes is plotted in Figure 3. It shows an exponential decay relationship, and the surface roughness of 0.15 nm almost reaches the minimal limit.

During the process, we find that some silica nanoparticles in the abrasive slurry adhere to the chip surface, seen in the above AFM images, which cannot be removed by ultrasonic cleaning or by HF acid. It is because an amorphous SiC thin film, generated during polishing, covers the whole surface of these nanoparticles, which prevents them from dissolving in the HF acid. In order to totally remove these nanoparticles, a post low-temperature thermal oxidation is carried out to oxidize the amorphous SiC, so that it can be successfully dissolved in HF acid. The AFM image of the final surface profile of the 4H-SiCOI chip is shown in Figure 2c. The smooth surface does not have any abrasive nanoparticle on top.

## 3. Device Fabrication and Characterization

We fabricate the optical devices on two high-purity semi-insulating 4H-SiCOI chips, made by the ion-cut method, one is unpolished and the other runs thrice wet-oxidation-assisted CMP processes. The pattern is defined on the electron-beam (e-beam) resist (AR-P 6200.09), through resist spin coating, e-beam writing (JEOL JBX-9500FSZ), and developing. Then, the pattern is transferred to the aluminum layer, used as a hard mark to etch 4H-SiC, through the e-beam metal evaporation and the lift-off processes. Next, the pattern is transferred to the 4H-SiC layer, by inductively coupled plasma-reactive ion etching (ICP-RIE). Finally, a silicon oxide layer is deposited on top of the chip, by plasma enhanced chemical vapor deposition (PECVD). It is worth mentioning that all these fabrication processes for making the 4H-SiCOI integrated photonic devices are CMOS compatible.

**Figure 4:**
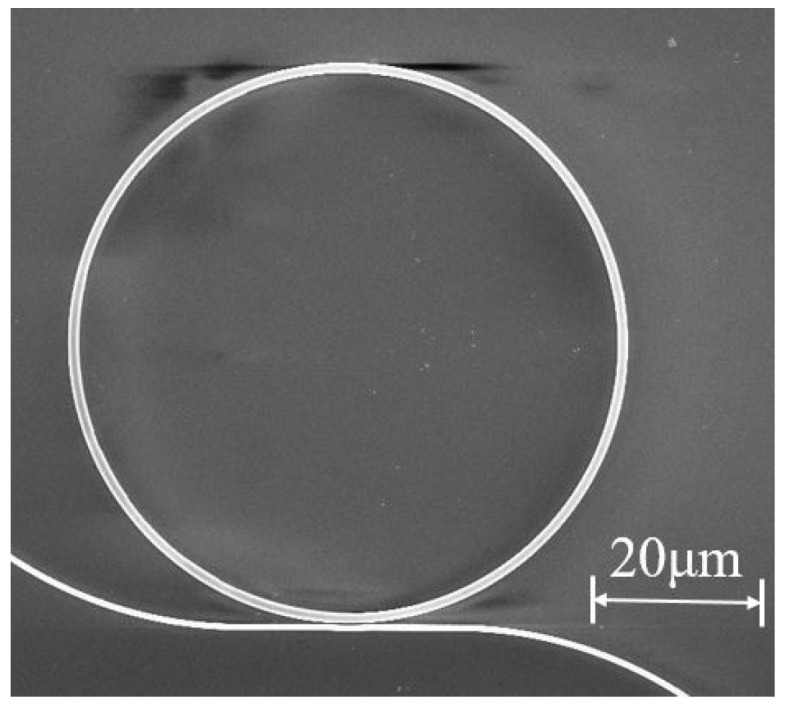
Top-view SEM image of the fabricated microring resonator in the 4H-SiCOI chip.

Figure 4 shows a scanning electron microscope (SEM) image of the fabricated microring resonator in the 4H-SiCOI chip. The microring has a cross-section dimension of 400 nm × 1200 nm and a diameter of 66 µm. To characterize microring resonators, a tunable continuous-wave (CW) laser is used as the light source. The light is launched into a polarization controller, to adjust the polarization to be transverse-electric mode, and is coupled in and out of waveguide from the edge of the chip through a pair of lensed fibers. The output light is detected by an optical spectrum analyzer, which is synchronized with the tunable CW laser.

**Figure 5:**
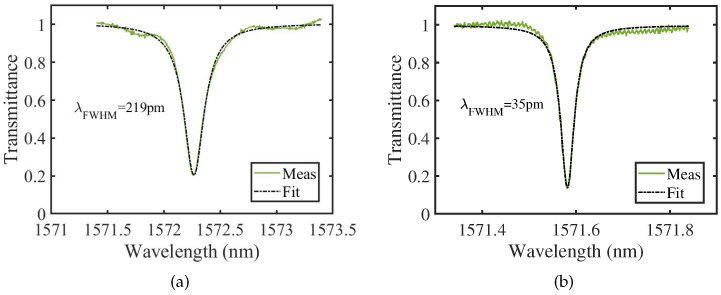
Transmittance of the optical microring resonators that are fabricated in the (**a**) unpolished and (**b**) polished 4H-SiCOI chips.

We measure the transmission spectrum of the resonance of the microrings fabricated in both unpolished and polished 4H-SiCOI chips at around 1572 nm, which is shown in Figure 5a and Figure 5b, respectively. The optical propagation loss in the waveguide can be extracted from the resonance spectrum of the microring resonators [31]. The quality factor of the microring can be calculated by

(1) $$Q=\frac{\lambda }{\lambda_{FWHM}},$$

where λ is the resonant wavelength, and $\lambda_{FWHM}$ is the full-width at half-maximum (FWHM) of the resonance. The quality factor is related to the intrinsic quality factor, $Q_{in}$, and the external quality factor, $Q_{ex}$, expressed as

(2) $$Q=\frac{Q_{in}Q_{ex}}{Q_{in}+Q_{ex}}.$$

The extinction ratio is also related to the intrinsic quality factor and the external quality factor, expressed as

(3) $$EX={\left|\frac{Q_{in}-Q_{ex}}{Q_{in}+Q_{ex}}\right|}^{2}.$$

From the measurement, we are able to extract the FWHM of the resonance and the extinction ratio directly from the resonant spectrum. Thus, both the intrinsic quality factor and the external quality factor can be obtained, accounting for the loss and the coupling, respectively.

After fitting the measured data with Lorentzian curves in Figure 5, the FWHM is found to be 219 pm and 35 pm, and the corresponding quality factor is calculated as 7.2 $\times 10^{3}$ and 4.49$\times 10^{4}$ according to Equation (1), for the unpolished and polished 4H-SiCOI chips, respectively. With the measured extinction ratio, the intrinsic quality factor is calculated as 9.6 $\times 10^{3}$ and 6.20 $\times 10^{4}$, in the unpolished and polished 4H-SiCOI chips, through Equations (2) and (3). It is seen that the intrinsic quality factor of the polished 4H-SiCOI chip is increased by a factor of 6.5. For the polished chip, the loss is mainly induced by the sidewall roughness of the waveguides and the material absorption because of the defects in the crystal generated during the ion implantation. The loss induced by the rough sidewalls could be potentially reduced by optimizing the dry etching condition to have a smoother sidewall.

## 4. High-Temperature Thermal Annealing

High-temperature annealing has been predicted to effectively recover the defects in the hydrogen ion implanted 4H-SiC thin film, and thus can help to reduce the light propagation loss induced by the material absorption [12]. We anneal the polished 4H-SiCOI chip at 1300 °C for 3 h in a high temperature, inductively heated graphite furnace [32]. The furnace was first heated up to 1000 °C under a vacuum of 1.5 $\times 10^{-4}$ mbar and then filled with high purity Ar to 550 mbar once the temperature is reached. A heating rate of 5 °C/min has then been applied up to 1300 °C. The furnace is then naturally cooled, with a slow cooling rate as it has a high thermal inertia.

**Figure 6:**
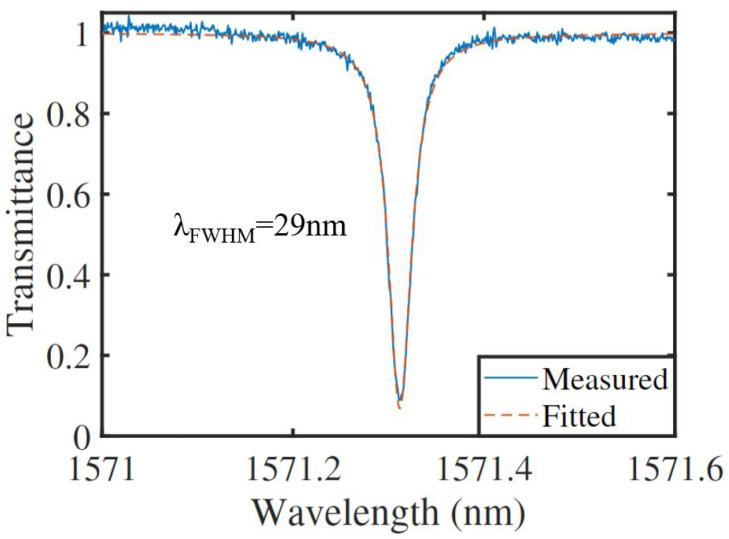
Transmittance of the optical microring resonators after high temperature annealing.

We then characterize same microring resonator fabricated in the high-temperature annealed 4H-SiCOI chip, shown in Figure 6. The intrinsic quality factor is extracted to be 8.17 $\times 10^{4}$. Compared to the performances of the 4H-SiC microring resonator without annealing, the intrinsic quality factor is increased by a factor of 1.3.

Additionally, we also tried higher temperature annealing at 1390 °C, but the 4H-SiC was delaminated from the substrate, due to the large thermal mismatch between 4H-SiC and the substrate. The annealing temperature of 4H-SiCOI is also limited by the melting point of Si, which is about 1410 °C. Provided the bonding strength is improved, the materials, such as sapphire, with much higher melting point can be used as the substrate.

## 5. Conclusions

In conclusion, we report two effective methods to reduce the light propagation loss in the 4H-SiCOI integrated platform, fabricated through the ion-cut method. A wet-oxidation-assisted chemical mechanical polishing method is proposed to polish 4H-SiC, which does not use any oxidizing or corrosive wet chemicals and is environmentally friendly. With three cycles of processing, the surface roughness of the 4H-SiCOI chip is decreased greatly from 3.67 ± 0.23 nm to 0.15 ± 0.04 nm. We experimentally demonstrate that the intrinsic quality factor of the microring resonator in the polished 4H-SiCOI chip is significantly increased to 6.20 $\times 10^{4}$. We also demonstrate that high-temperature thermal annealing at 1300 °C can further reduce the loss of the ion-implanted 4H-SiCOI chip, and finally achieve a higher intrinsic quality factor of 8.17 $\times 10^{4}$. The proposed methods can also be applied to SiC of different polytypes for low-loss photonic integrated circuits.

## Data Availability

The data presented in this study are available on request from the corresponding author.
